# A New Treatment for Chronic Dysentery

**Published:** 1869-02

**Authors:** E. Malcolm Morse

**Affiliations:** San Francisco


### A NEW TREATMENT FOR CHRONIC DYSENTERY.

By E. MALCOLM MORSE, M.D., San Francisco.

Chronic Dysentery generally means inflammation and ulceration of the large intestine. Instead of expecting a cure by giving medicines by the mouth, to act through the blood, or to travel ten yards before arriving at the seat of the disease, or giving medicated enemas in so small a bulk that they are hardly sufficient to fill the rectum, I have been in the habit of washing out the whole rectum and colon by throwing up into the large intestine from two to five pints of Labarraque's solution of the chloride of soda, diluted, thus making a topical application to the ulcers of one of the best, most cleansing, stimulating, and healing solutions contained in our Pharmacœpia. This remedy gives little or no pain, is perfectly safe, and may be considered a specific in uncomplicated ulceration of the large intestine. By uncomplicated ulceration of the large intestine, I mean dysentery not kept up by the organic disease of the heart, or phthisis pulmonalis; and not dependent on irremediable obstruction of the liver or spleen. For in each of these four cases the dysentery is produced by, or complicated with, a more serious primary disease.

In presenting to the members of the Medical profession, this plan of treatment for chronic dysentery, not found in any of the text books with which I am familiar, I would feel some hesitation, and not a little responsibility, did I not know that the theory itself is based on rational principles; and the success of the application of this theory as witnessed by myself during eleven years, is so marked that I can confidently recommend it as a safe cure for some of the worst cases of this formidable disease. I have seen patients that have been suffering for months and even years, with chronic dysentery, rescued by the application of these chloride of soda enemas, from the jaws of death.

The mortality in chronic dysentery, both under the old and the latest method of treatment, is very great.

On examination of the bellies of those who have died with this disease we find the mucous membrane of the large intestine extensively ulcerated; very often ulcers are low down; they are found principally in the rectum and descending colon; often in the tranverse colon and cæcum. Dr. Wood says: "The mucous membrane of the rectum and the lower portion of the colon always evince signs of inflammation in cases of death by dysentery. It is much reddened and thickened and not unfrequently ulcerated. Ulcers, in fact, exist in this disease more frequently than in any other acute inflammation of the alimentary canal, unless in the follicular enteritis of typhoid fever and small-pox. The danger is proportionate to the extent of the colon involved." Now, if we have a patient suffering from a simple ulcer in the mouth, we do not attempt to heal it by throwing up astringent or opiate enemas into his rectum; we apply the remedial agent directly to the seat of the injury. And if we have a patient with ulcers in the colon, why not apply the proper medicine at once to the proper place?

In order to get the patient into a proper condition to derive the most benefit from these injections, I am in the habit of pursuing the following method. I regulate his diet carefully, of course, and keep him in a recumbent position in order to assist the return of blood from the engorged mesenteric veins, and those smaller tributaries which are distributed along the large intestine. This state of engorgement prevents the ulcers from healing, and renders each ulcer an outlet from which, in blood serum, the stream of life ebbs out like water from the tubs of the daughters of Danaus. At day-break on every alternative or fourth day, I give a mild cathartic or aperient, in order to clear out the alimentary canal. The ordinary contents of the intestine produce great irritation when it is in this engorged and hyperæsthetic condition; and it is better to get rid of the fæces about the same time, instead of letting them run in driblets over the raw surface every hour or two. After the cathartic or aperient has acted sufficiently, I inject very slowly from two to four pints of Labarraque's solution of chloride of soda, diluted, into the large intestine; in this way it becomes a topical application. The right strength for the first enema, is twenty parts of water to one of Labarraque's solution. I inject as much of this mixture as he can be made to retain. Two or three pints will generally be enough. Sometimes as much as five pints may be given. Each enema should be a little stronger, until the patient can feel it smart or burn. When this happens the solution is of the proper strength. The patient should be on his right side, or on his knees with his head low down, while these enemas are being administered. Occasionally it is necessary for him to change his position several times, in order that the wash may reach every point where it is needed. Should there be much tenesmus after the injection has been passed, I give an enema of the tinct. opii, or an opium suppository. These applications of the chloride of soda should generally be made once a day. Occasionally it is necessary to give them twice a day; and sometimes on account of the sensitiveness of the ulcers as they begin to heal, it is better to leave them off for several days, or give weaker solutions. The next indication in the treatment, after cleaning out the alimentary canal and washing the ulcers with the medicated solution, is to keep the bowels quiet, so that the ulcers may remain clean and heal up under the topical application. In suggesting the means of accomplishing this desideratum, I am getting upon very debatable ground. The old proverb, "tot hominies tot sententiones" must certainly have been arms intended for physicians. Each one of us has his own way of using the with which we combat disease. I generally give large doses of subnitrate of bismuth, three times a day; repeated opiate enemas and suppositories, in order not to disorder the stomach; Dover's powders, repeated if necessary; charcoal, or the mineral and vegetable astringents; the ant-acids, leeches and foementations; taking great care to *keep up* the effect of the medicine, by giving them every hour or two. If one drug fails I try another, or give a combination of several of them; in order to have as few stools as possible passing over the ulcerated surfaces while they are healing.—*California Medical Gazette.*

---

SPIROGRAPH.—We learn from the *Lancet*, that Dr. David C. McVail, of Northumberland, has constructed an instrument which, as he claims, will record the respiratory movements accurately, and can be applied easily to any portion of the chest or abdomen.—*Med. and Surg. Reporter.*

